# The impact of food fortification on stunting in Zimbabwe: does gender of the household head matter?

**DOI:** 10.1186/s12937-020-00541-z

**Published:** 2020-03-23

**Authors:** Terrence Kairiza, George Kembo, Asankha Pallegedara, Lesley Macheka

**Affiliations:** 1 Department of Economics, Bindura University of Science Education, P. Bag 1020, Bindura, Zimbabwe; 2 Food and Nutrition Council of Zimbabwe, 1574 Alpes Road, Hatcliffe, Harare, Zimbabwe; 3 Department of Industrial Management, Wayamba University of Sri Lanka and Chair of Development Economics, Passau University, Passau, Germany; 4 Centre for Innovation and Technology Transfer, Marondera University of Agricultural Sciences and Technology, P. Bag 35, Marondera, Zimbabwe

**Keywords:** Child stunting; gender, Biofortification, Fortification, Micronutrient deficiency

## Abstract

**Background:**

High prevalence of stunting in children under 5 years poses a major threat to child development in developing countries. It is associated with micronutrient deficiency arising from poor diets fed to children under 5 years. Food fortification is amongst the interventions focused at reducing the incidence of stunting in children under 5 years.

**Methods:**

Using a large-scale household data from Zimbabwe, we investigated the gender-based importance of household adoption of food fortification on the proportion of stunted children in the household. We employed propensity score matching to mitigate self-selection bias associated with household adoption of food fortification.

**Results:**

We offer three major findings. Firstly, we find statistically weak evidence that female headed households are more likely to adopt food fortification than their male counterparts. Secondly, food fortification reduces the proportion of stunted children in the household. Finally, in comparison to non-adopters, female headed households that adopt food fortification are more able to reduce the proportion of stunted children in their households than their male counterparts.

**Conclusion:**

The results highlight the need for policy makers to actively promote food fortification, as such interventions are likely to contribute to the reduction of stunting and to involve men in fortification interventions to improve on their knowledge and appreciation of fortified foods and the associated benefits.

## Background

The preponderance of stunting (anthropometric indicator, height-for-age z-score, HAZ < 2 standard deviations below the WHO International Growth Reference) in children under 5 years poses an intractable threat to child development in developing countries [34]. Stunting in children under 5 years is associated with elevated risk of child morbidity, mortality, as well as, poor cognitive and psychomotor development [20]. Long-term consequences of stunting include deficits in school achievement and work capacity [17]. Low micronutrient density and poor protein quality in cereal based diets availed to children under 5 years has been identified amongst the preeminent causes of stunting in resource poor settings. Whilst the prevalence of stunting in children under five has been decreasing worldwide over the past two decades, [23] estimated that globally, at least 165 million children under 5 years were stunted in in 2011. In Zimbabwe, the proportion of children under 5 years who were stunted in 2018 was 26% representing a decline from 34% recorded in 2010 [35]. The 2018 stunting rate of 26% in Zimbabwe however still falls short of the acceptable target of 20% by UNICEF. Iron deficiencies among children under 5 years is exceedingly high, estimated at 72% and anemia is also prevalent in this age group [25]. Iron deficiency is even higher among infants of 6 to 11 months (81%), hence micronutrient stores among infants are a major determinant to stunting in the Zimbabwean setting. Poor complementary diets after 6 months indicate that the entire 1000 days the Zimbabwean child thrives in an environment that lacks iron, calcium, vitamin A, and high-quality protein [25]. These are all linked to stunting in the Zimbabwean setting.

Interventions aimed at ameliorating the low micronutrient intake in children under 5 years include the promotion of household adoption of food fortification, which can be industrial fortification (adding micronutrients and minerals to industrially processed and widely consumed edible products), supplementation (addition of an essential micronutrient during food preparation) or biofortification (improving the nutritional quality of food crops through agronomic practices, conventional plant breeding, or modern biotechnology). In June 2017, the Government of Zimbabwe made it mandatory for major local food manufacturers to fortify processed staple foods with micronutrients. The food vehicles targeted for fortification included sugar (vitamin A), cooking oil (vitamin A and D), maize meal and wheat flour (A, B1, B2, B3, B6, B12, folic acid, iron and zinc). In addition to mandatory fortification, the Government of Zimbabwe is promoting three biofortified food crops which are, biofortified orange maize (Vitamin A), Nua 45 beans (Zinc and Iron), and protein maize.

Notwithstanding the efforts to improve the adoption of food fortification, in Zimbabwe and in developing countries in general is still low [33]. Adoption of food fortification at the household level is confounded by a host of systemic and idiosyncratic factors, including the gender of the household head. Sachs (1996) [31] and Quisumbing et al. [27] note that while women are typically responsible for the preparation of the food for children under 5 years, men tend to exercise control over the economic availability of the food which points to the need to incorporate household head gender into studies that seek to identify the impact of food fortification on stunting. Identification of the impact of food fortification using observational data is confounded by self-selection bias associated with household adoption of food fortification [5, 6, 18]. Randomized controlled trials circumvent the self-selection bias due to the exogenous assignment of households into treatment and control group [18]. The findings of randomized controlled trials on the impact of household adoption of food fortification on stunting and other child development outcomes is however ambivalent [13]. The reason for the inconclusive results could be due to the fact that the pathogenesis of stunting is poorly understood. Furthermore, to the best of our knowledge, extant studies have not incorporated heterogeneity in the impact food fortification on the basis of the gender of the household head who determines both access and preparation of fortified foods in the household.

The main aim of this study was to investigate the impact of food fortification on the basis of gender of the household head, who usually determines both access and preparation of fortified foods in the household. We address the aforementioned gaps in the literature by examining the gender attributes of the impact of household adoption of food fortification on the proportion of stunted children in the household using the 2018 nationally representative sample of 25,297 Zimbabwean households surveyed by the Food and Nutrition Council of Zimbabwe (FNC). We measure the adoption of food fortification using five proxies that indicate both knowledge and usage of food fortification. To identify the average treatment effect of household adoption of fortified foods on the proportion of stunted children under 5 years, we employ propensity score matching techniques to counter the self-selection bias associated with the household adoption of food fortification [3].

### Hypotheses

The high incidence and grievous consequences of childhood undernutrition in sub-Saharan Africa necessitated emphasis on early prevention [26]. Food fortification, in its three forms: industrial fortification, supplementation fortification, and biofortification is one of the strategies that has been used to prevent vitamin and mineral deficiencies [8]. Notwithstanding the efforts to improve the adoption of food fortification in Zimbabwe and in developing countries in general is still low [33]. We therefore propose the following hypothesis linking the gender of the household head and the household probability of adopting food fortification.

#### Hypothesis 1

Female headed households are more likely to adopt food fortification than their male counterparts.

Several efficacy and impact studies have shown that food fortification in its three forms can have a nutritional impact [7, 9, 12, 14, 22]. For example, studies conducted in rural Uganda showed that the introduction of Orange-fleshed sweet potato (OFSP) resulted in increased vitamin A intakes among children and women, and improved vitamin A status among children [19]. An efficacy study conducted in Zambia with 5–7-year-old children showed that, after 3 months of consumption of biofortified provitamin A, the total body stores of vitamin A in the children who were in the orange maize group increased significantly compared with those in the control group [15]. Results from community-based, randomized controlled supplementation trials (zinc, iron and vitamin A) [29] showed that provision of iron supplements to the anemic infants or young children resulted in improved growth. Furthermore, vitamin A supplementation had a significant positive effect on stunting reduction in subgroups of children of low socioeconomic status. In this background we therefore propose the following hypothesis linking the adoption of food fortification and the proportion of stunted children in the household.

#### Hypothesis 2

Household adoption of food fortification reduces the proportion of stunted children in that household.

Although there is burgeoning evidence on the impact of food fortification on stunting, research on gender heterogeneity in the impact of food fortification on stunting is still limited. Several studies have shown that increasing women’s bargaining power is associated with improved child outcomes, e.g. reduced stunting [4, 28]. Richards et al. [28] discussed the significant and positive nutritional outcomes relating to women’s household authority. Intra-household gender dynamics regarding decisions about crop choice and child-feeding practices have proven to play a role in adoption decisions. It is likely that if women are household heads, they are likely to have higher impact of food fortification since they are also largely responsible for food preparation in the household [27, 31]. We therefore propose the following hypothesis linking the gender of the household head and the impact of food fortification on the proportion of stunted children in the household.

#### Hypothesis 3

Female headed households that adopt fortification are more able to reduce the proportion of stunted children in their households than their male counterparts.

## Methodology

### Study design

The data employed herein stems from the 2018 Zimbabwe National Nutrition Survey (NNS) which was carried out by the Food and Nutrition Council of Zimbabwe (FNC) supported by the multisectoral National Nutrition Survey Technical Committee (NNSTC). NNSTC is a consortium of Government Ministries, UN partners, Technical Organisations and NGOs. The data comprises of a sample of 25,297 households with at least one child under 5 years. The sample households are randomly drawn from the sampling frame of the 2012 National Census so that they are representative of the national population of households with children under 5 years.

### Measurement of key variables

#### Proportion of stunted children in the household

Height for children under 5 years was measured during the survey using standard equipment and methods. Children younger than 24 months were measured lying down, whilst children between 24 and 59 months were measured whilst standing. Anthropometric index height for age Z-score (HAZ) was analyzed using WHO Anthro software version 3.2.2. A child under 5 years is categorized as stunted if HAZ < − 2 standard deviations below the WHO International Growth Reference.

Our analysis seeks to identify the drivers of stunting at the household level, our outcome of interest is therefore the proportion of stunted children in the household which is measured as follows:

Proportion of stunted children in the household *i* = Number of children under 5 years with HAZ < 2 in household *i* / Number of children under 5 years in household *i*.

#### Household adoption of food fortification

The 2018 Zimbabwe National Nutrition Survey asked five questions that relate to the adoption and knowledge of food fortification. We use these five questions as proxies for the household adoption of food fortification. Firstly, the survey asked whether the household head had ever heard about fortified foods. Secondly, it asked whether the household head is able to identify fortified foods in the market. Thirdly, it asked whether the household had purchased any fortified food product in the past 30 days. These three questions relate to industrial fortification. Fourthly, the survey asked whether the household had fed an under 5 child meals containing micronutrient powders in the past 30 days, relating to supplementation. Finally, the survey asked whether the household head had ever heard about biofortified crops. The final question relates to biofortification adoption. The five proxies of fortification take the value of 1 if the household head answered *Yes* and, 0 otherwise.

#### Other control variables

The survey also asked other questions pertaining to the socio-demographic characteristics of the household head as well as the household, among which are the gender, age, marital status and education of the household head. Household level control variables include, household size, proportion of economically active household members, number of children with chronically or mentally ill mothers or fathers, as well as whether the household is located in rural areas. We also control for the province where the household is resident.

### Empirical estimation

To test Hypothesis 1 of this study, we employ binary response models to estimate the impact of the gender of the household head on the probability of the household adoption of food fortification and present the results in Table 4. Assessing the impact or the treatment effect of food fortification on stunting using observational data as ours, is confounded by incomplete information arising from the self-selection of observations into adopting food fortification [18]. We therefore employ Propensity Score Matching (PSM) to eliminate the self-selection bias. Using PSM, we can reduce or eliminate the problem of systemic differences in baseline characteristics between treated and untreated groups [18].

We define an indicator variable, Fort_i_, which takes the value of 1 for household *i*, if the household adopted food fortification, and 0 otherwise. We also define the dependent variable, the proportion of stunted children in household *i* as Y_i_. The counterfactual problem is that for each household we can only observe either Y_i1_, or Y_i0_ which are the proportion of stunted children in the household given Fort_i_ = 1 and Fort_i_ = 0, respectively.

Propensity score matching techniques circumvent the counterfactual problem by matching Fort_i_ = 1 and Fort_i_ = 0 households using Pr (Fort_i_ = 1| **X**) which is the probability of household *i* having Fort_i_ = 1 on the basis of observed covariates, **X**. In this study, we use nearest neighbour matching technique which chooses an individual from the comparison group for treated individual that is closest in terms of propensity score. We estimate the average treatment effect on the treated (ATT) that provides the impact of food fortification on the proportion of stunted children in the household as follows:

1 $$ \mathrm{ATT}=\mathrm{E}\ \left({\mathrm{Y}}_{\mathrm{i}1}\ |\ {\mathrm{Fort}}_{\mathrm{i}}=1\right)-\mathrm{E}\ \left\{\mathrm{E}\ \left({\mathrm{Y}}_{\mathrm{i}0}\ |\ {\mathrm{Fort}}_{\mathrm{i}}=0,\Pr\ \left({\mathrm{Fort}}_{\mathrm{i}}=1|\mathrm{X}\right)\ |{\mathrm{Fort}}_{\mathrm{i}}=1\right)\right\} $$

We employ the user written Stata module PSMATCH2 developed by Leuven and Sianesi [21] to implement matching and estimate treatment effects and present the results in Table 5 which portray the test to Hypotheses 2.

To test Hypotheses 3 which examine gender heterogeneity in the impact of Fort_i_ = 1 on the basis of the gender of the household head, Female_i_, which takes the value of 1 if the household head is female and 0 otherwise we separately estimate ATT presented in Eq. 1 given that Female_i_ = 1 and Female_i_ = 0 and present the results in Tables 6.

The validity of the ATT requires the Conditional Independence Assumption (CIA), that assignment to Fort_i_ = 1 or Fort_i_ = 0 is random after controlling for observed covariates **X** [18]. We perform a balance of covariates before and after propensity score matching as robustness check and present the results in Table 7.

## Results and discussion

### Descriptive analysis

#### Differences in background characteristics by food fortification adoption status of the household

On the basis of whether the household head had ever heard about fortified foods, Table 1 shows the differences in the background characteristics of the sample households by the food fortification status of the household. The table shows that out of the 25,297 households with children under 5 years that were surveyed, 3038 (12%) knew fortified foods. These households are taken to have adopted food fortification. Furthermore, the table reveals that households that had adopted food fortification are less likely to be female headed than those that had not adopted food fortification by 1.9% at the 5% level of significance, before controlling for observed confounders.

**Table 1:** Background characteristics of households by treatment status

Variable	Adopted Food Fortification				Difference in means [Y – N]
	Yes [Y]		No [N]		
	Mean	SD	Mean	SD	
Observations (%)	3038	(12%)	22,259	(88%)	
Household head is female	0.246	0.43	0.265	0.441	−0.019^b^
Household head age [Years]	42.329	14.912	42.266	15.637	0.063
**Marital status of household head**					
Married	0.811	0.392	0.791	0.407	0.020^a^
Living with partner	0.022	0.146	0.021	0.142	0.001
Divorced/separated	0.034	0.18	0.047	0.212	−0.014^a^
Widow/Widower	0.12	0.325	0.129	0.335	−0.009
Never married/never lived with a partner	0.014	0.116	0.012	0.111	0.001
**Education level of household head**					
None	0.089	0.285	0.123	0.328	−0.034^a^
Primary level	0.311	0.463	0.406	0.491	−0.095^a^
ZJC level	0.108	0.31	0.098	0.297	0.010^c^
O′ level	0.396	0.489	0.336	0.472	0.060^a^
A’ level	0.023	0.151	0.017	0.131	0.006^b^
Diploma/Certificate after primary	0.008	0.091	0.003	0.051	0.006^a^
Diploma/Certificate after secondary	0.041	0.197	0.012	0.107	0.029^a^
Graduate/Post-Graduate	0.024	0.152	0.006	0.078	0.018^a^
**Household characteristics**					
Household size (number of members)	4.779	1.931	4.697	1.934	0.083^b^
Proportion of economically active household members	0.447	0.246	0.424	0.249	0.023^a^
Number of chronically ill household members	0.155	0.476	0.146	0.458	0.009
Number of mentally ill household members	0.084	0.323	0.091	0.34	−0.008
Number of children with sick mothers in the household	0.021	0.165	0.028	0.196	−0.007^b^
Number of children with sick fathers in the household	0.016	0.151	0.018	0.161	−0.001
Household is located in rural areas	0.927	0.26	0.925	0.264	0.002
**Province**					
Bulawayo	0.006	0.079	0.018	0.132	−0.011^a^
Manicaland	0.124	0.329	0.101	0.301	0.023^a^
Mash Central	0.118	0.322	0.118	0.322	0
Mash East	0.152	0.359	0.126	0.332	0.025^a^
Mash West	0.1	0.3	0.117	0.322	−0.018^a^
Mat North	0.184	0.388	0.109	0.312	0.075^a^
Mat South	0.088	0.283	0.102	0.303	−0.015^a^
Midlands	0.087	0.282	0.14	0.347	−0.053^a^
Masvingo	0.095	0.293	0.132	0.339	−0.037^a^
Harare	0.047	0.211	0.036	0.187	0.011^a^

Notes

Sample size of 25,297 is the actual number of households with children under 5 years

The fifth column shows the results of two-tailed t-test for the difference in the means. ^a^, ^b^, and ^c^ indicate the 1, 5, and 10% levels of significance, the assumption is that the sub-samples have an unequal variance

The data are expressed as proportions unless otherwise stated

Table 1 further reveals that households that adopted food fortification tend to be more educated than those that had not adopted food fortifications. Specifically, 39.6% of the households that adopted food fortification achieved O′ level education versus 33.6% of the households that had not adopted food fortification. This finding seems reasonable given that those household heads that are more educated are likely to have acquired knowledge about fortification through education. Furthermore, households that adopted food fortification, tend to be larger and have more economically active members than those households that did not adopt food fortification. Moreover, save for Mash Central there are statistically, significant province differences in the food fortification adoption status of the household. The differences in the background characteristics between those that adopted food fortification and those households that did not adopt point to self-selection bias in the adoption of food fortification (e.g., Heckman et al. [18]).

#### 3.1.2. Gender differences food fortification adoption

Table 2 shows that in comparison to male headed households, female headed households were less likely to have heard about fortified foods, identify them on the market or purchased them in the past 30 days of the survey. There is however no statistically significant gender difference in the probability of having fed an under 5 child meals with micronutrient powders in the past 30 days. Moreover, the table also shows that only 4.5% of female household heads had heard about biofortified crops versus the 5.6% of male household heads. In summary, the findings presented in Table 2 show that whilst females are less likely to have adopted food fortification than their male counterparts before controlling for other confounders. Furthermore, knowledge and usage of food fortification is generally low in Zimbabwe as only 11.2% of females and 12.3% of male household heads had heard about fortified foods. These results are consistent with the findings of Talsma et al. [33] who also reported low (below 15%) knowledge and usage of fortified foods in Benin, Brazil, Nigeria and South Africa.

**Table 2:** Uptake of fortified foods by gender of the household head

Type of Food Fortification	Proxy of Food Fortification Adoption	Female [F]		Male [M]		Difference in means [F – M]
		Mean	SD	Mean	SD	
	Observations # (%)	7017	(27.7%)	18,280	(72.3%)	
Mandatory	Ever heard about Fortified foods [1 if Yes, 0 if No]	0.112	0.316	0.123	0.328	−0.010^b^
	Able to identify fortified foods on the market [1 if Yes, 0 if No]	0.105	0.306	0.118	0.322	−0.013^a^
	Purchased any fortified food product in the past 30 days [1 if Yes, 0 if No]	0.152	0.359	0.179	0.384	−0.027^a^
Supplementation	Fed child (6–23 months) meals with micronutrient powders in the past 30 days [1 if Yes, 0 if No]	0.046	0.209	0.049	0.216	−0.003
Biofortification	Ever heard about Bio fortified Crops [1 if Yes, 0 if No]	0.045	0.207	0.056	0.229	−0.011^a^

Notes: Sample size is 25,297. The fifth column shows the results of two-tailed t-test for the difference in the means. ^a^, ^b^, and ^c^ indicate the 1, 5, and 10% levels of significance

#### Proportion of stunted children

Table 3 shows the proportion of stunted children in the household by the gender of the household head as well as the food fortification adoption status. The table reveals that there is no statistically significant gender difference in the proportion of household heads that adopted food fortification. When looking at the subsample of female headed households, those who adopted food fortification have a lower proportion of stunted children under 5 years of 24.2% in comparison to the 30.2% for those female headed households who did not adopt food fortification. Furthermore, the difference of 6% is statistically valid at the 1% level of significance. The respective proportions for the male headed households are 25.7 and 29.0% establishing a difference of 3.3%.

**Table 3:** Proportion of stunted children in household by gender and food adoption status

		Household head gender:			Difference [F – M]
		Total	Female [F]	Male [M]	
Household adopted food fortification	Yes [Y]	0.253	0.242	0.257	−0.015
	No [N]	0.294	0.302	0.290	0.012^c^
Difference in means	[Y – N]	−0.040^a^	− 0.060^a^	− 0.033^a^	− 0.027

Notes: Sample size is 25,297. The fifth column shows the results of two-tailed t-test for the difference in the means. ^a^, ^b^, and ^c^ indicate the 1, 5, and 10% levels of significance

The sum total of these findings is that adoption of food fortification is correlated with reduction of stunting and furthermore, female who adopt fortification are more able to reduce stunting than their male counterparts, before controlling for self-selection bias associated with adoption of food fortification. However, it is important to control for the self-selection bias to estimate true relationship between food fortification and reduction of child stunting.

### Estimation results

#### The impact of gender on the adoption of fortification

Table 4 shows the probit estimates of the marginal effects of the gender of the household head on the adoption of food fortification. Columns (I) to (III) of the table indicate no statistically significant impact of household head gender on the probability of ever having heard of fortified foods, being able to identify fortified foods on the market or purchasing any fortified foods in the past 30 days. Columns (IV) and (V) of the table displays statistically weak evidence of female household heads having a fed an under 5 child meals with micronutrient powders in the past 30 days or having have ever heard about biofortified crops. The table reveals that rather than gender, the most important variable determining the adoption of food fortification is education of the household head. Columns (I) to (V) of Table 4 show that in compared to base uneducated household heads, attaining any level of education increases the probability of adopting food fortification. Moreover, the impact of education on the probability of adopting food fortification increases as the level of education increases. This result is consistent with earlier studies such as [1, 2, 10, 16, 24] which reported significant adoption of fortified foods by mothers who had secondary/tertiary education in comparison with uneducated mothers.

**Table 4:** Probit estimates of the impact of gender on the adoption of fortified foods

	Proxy of Food Fortification Adoption:				
	Ever heard about Fortified foods [1 if Yes, 0 if No]	Able to identify fortified foods on the market [1 if Yes, 0 if No]	Purchased any fortified food product in the past 30 days [1 if Yes, 0 if No]	Fed child (6–23 months) meals with micronutrient powders in the past 30 days [1 if Yes, 0 if No]	Ever heard about Bio fortified Crops [1 if Yes, 0 if No]
	(I)	(II)	(III)	(IV)	(V)
Household head is female	0.00764	−0.00107	0.00110	0.00892^b^	0.00830^c^
	(0.00678)	(0.00778)	(0.00650)	(0.00445)	(0.00447)
Household head age [Years]	0.000349^b^	0.000325	0.000557^a^	−4.20e-05	0.000617^a^
	(0.000169)	(0.000203)	(0.000166)	(0.000104)	(0.000103)
Married	−0.0185	− 0.0272	− 0.0554^b^	− 0.0111	0.0120
	(0.0204)	(0.0271)	(0.0217)	(0.0145)	(0.0134)
Living with partner	−0.00669	− 0.0324	− 0.0170	− 0.0166	− 0.00523
	(0.0222)	(0.0267)	(0.0193)	(0.0109)	(0.0166)
Divorced/Separated	−0.0379^b^	0.00301	−0.0334^b^	−0.0137	0.00533
	(0.0163)	(0.0278)	(0.0154)	(0.0104)	(0.0178)
Widow/Widower	−0.0131	−0.0197	− 0.0337^b^	− 0.0177^c^	− 0.00332
	(0.0188)	(0.0247)	(0.0154)	(0.00979)	(0.0151)
Primary level	0.0123	0.0167^c^	0.0501^a^	0.00129	0.0135^b^
	(0.00757)	(0.00919)	(0.00842)	(0.00487)	(0.00525)
ZJC	0.0546^a^	0.0754^a^	0.0949^a^	0.0180^b^	0.0332^a^
	(0.0117)	(0.0135)	(0.0137)	(0.00746)	(0.00896)
O′ level	0.0770^a^	0.0715^a^	0.111^a^	0.0162^a^	0.0379^a^
	(0.00916)	(0.0105)	(0.0102)	(0.00564)	(0.00647)
A’ level	0.120^a^	0.111^a^	0.169^a^	0.0170	0.0502^a^
	(0.0251)	(0.0265)	(0.0282)	(0.0131)	(0.0183)
Diploma/Certificate after primary	0.270^a^	0.174^a^	0.299^a^	0.0615^c^	0.110^b^
	(0.0589)	(0.0587)	(0.0610)	(0.0350)	(0.0442)
Diploma/Certificate after secondary	0.329^a^	0.309^a^	0.379^a^	0.0674^a^	0.118^a^
	(0.0306)	(0.0319)	(0.0318)	(0.0201)	(0.0250)
Graduate/Post-Graduate	0.353^a^	0.300^a^	0.413^a^	0.0991^a^	0.201^a^
	(0.0395)	(0.0410)	(0.0400)	(0.0280)	(0.0372)
Household size	0.00387^a^	0.00488^a^	0.00337^a^	0.00140^c^	0.00170^b^
	(0.00116)	(0.00145)	(0.00116)	(0.000782)	(0.000729)
Proportion of economically active household members	0.0299^a^	0.103^a^	0.0625^a^	0.0291^a^	0.0231^a^
	(0.00867)	(0.0104)	(0.00855)	(0.00557)	(0.00548)
Number of chronically ill household members	0.00739^c^	−0.00165	−0.00229	0.0110^a^	0.00181
	(0.00448)	(0.00546)	(0.00457)	(0.00251)	(0.00292)
Number of mentally ill household members	− 0.00972	0.00388	− 0.00548	0.00597^c^	− 0.00404
	(0.00642)	(0.00719)	(0.00614)	(0.00357)	(0.00392)
Number of children with sick mothers in the household	−0.0168	0.00997	−0.0262^b^	− 0.00904	− 0.0117
	(0.0119)	(0.0130)	(0.0122)	(0.00735)	(0.00766)
Number of children with sick fathers in the household	0.00406	0.0233	0.00445	0.00833	0.00238
	(0.0132)	(0.0144)	(0.0123)	(0.00702)	(0.00771)
Household is located in rural areas	0.0351^a^	0.00445	0.0336^a^	0.00177	0.0180^a^
	(0.0116)	(0.0158)	(0.0114)	(0.00811)	(0.00689)
Bulawayo	−0.0896^a^	−0.154^a^	−0.0822^a^	− 0.0433^a^	− 0.0409^a^
	(0.00757)	(0.00364)	(0.00725)	(0.00155)	(0.00283)
Manicaland	−0.0201	−0.0723^a^	− 0.0441^a^	−0.0109	− 0.0155^c^
	(0.0161)	(0.0146)	(0.0130)	(0.00824)	(0.00882)
Mash Central	−0.0267^c^	−0.104^a^	− 0.0560^a^	−0.0318^a^	0.00306
	(0.0158)	(0.0126)	(0.0122)	(0.00528)	(0.0120)
Mash East	−0.0194	0.0381^c^	0.00932	−0.0320^a^	−0.0267^a^
	(0.0162)	(0.0223)	(0.0182)	(0.00544)	(0.00723)
Mash West	−0.0460^a^	−0.0210	− 0.0449^a^	−0.0348^a^	− 0.0350^a^
	(0.0136)	(0.0184)	(0.0130)	(0.00473)	(0.00558)
Mat North	0.0348^c^	−0.0779^a^	−0.0115	− 0.0328^a^	−0.0400^a^
	(0.0207)	(0.0147)	(0.0164)	(0.00522)	(0.00503)
Mat South	−0.0376^b^	−0.118^a^	− 0.0465^a^	−0.0416^a^	− 0.0402^a^
	(0.0148)	(0.0112)	(0.0131)	(0.00376)	(0.00470)
Midlands	−0.0663^a^	−0.141^a^	− 0.0745^a^	−0.0346^a^	− 0.0204^b^
	(0.0120)	(0.00950)	(0.0104)	(0.00504)	(0.00818)
Masvingo	−0.0588^a^	−0.0290	− 0.0473^a^	−0.0310^a^	− 0.0459^a^
	(0.0126)	(0.0181)	(0.0129)	(0.00548)	(0.00436)
Observations	24,730	22,701	24,407	24,176	24,790
Pseudo R^2^	0.0399	0.0712	0.0454	0.0430	0.0577

Notes: Sample size is 25,297. Robust standard errors in parentheses. ^a^, ^b^, and ^c^ indicate the 1, 5, and 10% levels of significance

#### Homogeneous treatment effects of food fortification on stunting

Table 5 shows the impact of the adoption of food fortification on the proportion of stunted children in the household. The table reveals that all five proxies of food fortification adoption reduces the proportion of stunted children in the household. Specifically, Column (I) of the table shows that having heard about fortification reduces the proportion of stunted children by 4.69%. Furthermore, Column (II) and (III) show that being able to identify fortified foods in the market or actually purchasing the fortified foods in the past 30 days reduces the proportion of stunted children in the household by 2.08 and 3.33%, respectively. Moreover Columns (IV) and (V) indicate that having fed an under 5 child meals with micronutrient powders in the past 30 days or having ever heard about bio fortified crops reduces the proportion of stunted children by 2.73 and 3.56%, respectively.

**Table 5:** PSM estimates of homogeneous treatment effects on stunting

Type of Fortification	Proxy of Food Fortification Adoption	
Industrial Fortification:	Ever heard about Fortified foods [1 if Yes, 0 if No]	−0.0469 ^a^
		(0.011324)
	Able to identify fortified foods on the market [1 if Yes, 0 if No]	−0.0208^b^
		(0.009955)
	Purchased any fortified food product in the past 30 days [1 if Yes, 0 if No]	− 0.0333^a^
		(0.011683)
Supplementation:	Fed child (6–23 months) meals with micronutrient powders in the past 30 days [1 if Yes, 0 if No]	−0.0273^a^
		(0.018255)
Biofortification:	Ever heard about Bio fortified Crops [1 if Yes, 0 if No]	−0.0356^b^
		(0.017038)

Notes: Sample size is 25,297. Robust standard errors in parentheses. ^a^, ^b^, and ^a^ indicate the 1, 5, and 10% levels of significance

The findings in Table 5 show that the adoption of food fortification reduces the proportion of stunted children in the household and confirms earlier results from both observational studies [29] and randomized controlled experiments [15]. The findings indicate that to reduce stunting governments should promote food fortification through projects that are being currently undertaken.

#### Gender heterogeneous treatment impacts of fortification on stunting

We explore potential heterogeneities in the impact of food fortification on the proportion of stunted children in the household by the gender of the household head and present the results in Table 6. Table 6 shows that when one looks at all measures of food fortification, the impact of food fortification on stunting is higher when the household head is female than when the household head is male. Specifically, purchasing any fortified food in the past 30 days reduces the proportion of stunted children by 6.02% when the household head is female versus the statistically insignificant impact when the household head is male. Furthermore, having heard biofortified crops reduces the proportion of stunted children by 9.42% when the household head is female versus the 4.15% when the household head is male. The findings presented in Table 6 imply that food fortification has higher impact in reducing the proportion of stunted children in the household when the household head is female rather than when he is male. These findings therefore imply when the household head is female, she is in charge of both the preparation and economic availability of the food for the children under 5 years which gives extra benefit to them.

**Table 6:** PSM estimates of gender heterogeneous treatment effects on stunting

Type of Fortification	Proxy of Food Fortification Adoption	Household head is Female	Household head is Male
		(I)	(II)
Industrial Fortification:	Ever heard about Fortified foods [1 if Yes, 0 if No]	− 0.0451^b^	− 0.0255^c^
		(0.02235)	(0. 01298)
	Able to identify fortified foods on the market [1 if Yes, 0 if No]	−0.0379^c^	− 0.0225^c^
		(0. 02060)	(0.01142)
	Purchased any fortified food product in the past 30 days [1 if Yes, 0 if No]	−0.0602^a^	−0.0187
		(0.02357)	(0.01336)
Supplementation:	Fed child (6–23 months) meals with micronutrient powders in the past 30 days [1 if Yes, 0 if No]	−0.0798^b^	− 0.0239
		(0.03762)	(0.0208)
Biofortification:	Ever heard about Bio fortified Crops [1 if Yes, 0 if No]	−0.0942^b^	−0.0415^b^
		(0.03576)	(0.01969)

Notes: Sample size is 25,297. Robust standard errors in parentheses. ^a^, ^b^, and ^c^ indicate the 1, 5, and 10% levels of significance

### Robustness checks to observed heterogeneity

Table 7 presents results from covariate balance tests to appraise the comparability of covariates before and after matching. *P*-values for the equality of means of covariates like household head is female, widow/widower, education dummies, proportion of economically active household member, household size as well as several province dummies are smaller than 0.05 before matching but larger than 0.1 after matching, indicating that covariates were unbalanced before matching but became balanced after matching. Failure to reject the hypothesis of joint equality of means after matching indicated by a *p*-value larger than 0.05, shows that covariates for households that adopted food fortification and those that did not adopt food fortification are drawn from comparable distributions [11]. Additionally, a mean absolute bias of 1.5% is far smaller than the 5% recommended to yield reliable estimates [30].

**Table 7:** Covariate balance check before and after propensity score matching

	Mean before matching			Mean after matching			% Bias Reduction
Variables	Treated	Controlled	*P*-value	Treated	Controlled	*P*-value	
Household head is female	0.241	0.266	0.004	0.241	0.225	0.185	40.6
Household head age [Years]	42.038	42.485	0.153	42.038	42.069	0.938	93.2
Married	0.806	0.794	0.151	0.806	0.81	0.677	62.3
Living with partner	0.024	0.02	0.131	0.024	0.026	0.661	57.1
Divorced/separated	0.042	0.045	0.437	0.042	0.038	0.487	−12.7
Widow/Widower	0.113	0.13	0.018	0.113	0.109	0.572	70
Primary level	0.319	0.405	0	0.319	0.326	0.579	91.8
ZJC level	0.109	0.098	0.069	0.109	0.107	0.86	86.6
O′ level	0.409	0.333	0	0.409	0.404	0.697	93.2
A’ level	0.024	0.017	0.006	0.024	0.026	0.602	69.9
Diploma/Certificate after primary	0.008	0.003	0	0.008	0.009	0.66	79.6
Diploma/Certificate after secondary	0.041	0.012	0	0.041	0.036	0.394	84.5
Graduate/Post-Graduate	0.026	0.006	0	0.026	0.027	0.932	98.2
Household size	4.721	4.714	0.872	4.721	4.716	0.933	30
Proportion of economically active household members	0.456	0.42	0	0.456	0.459	0.615	90.6
Number of chronically ill household members	0.135	0.15	0.128	0.135	0.135	0.975	97.4
Number of mentally ill household members	0.084	0.09	0.407	0.084	0.076	0.358	−36.3
Number of children with sick mothers in the household	0.019	0.028	0.015	0.019	0.016	0.578	76.6
Number of children with sick fathers in the household	0.017	0.017	0.876	0.017	0.014	0.465	− 416.9
Household is located in rural areas	0.921	0.924	0.487	0.921	0.92	0.92	80.3
Bulawayo	0.008	0.019	0	0.008	0.006	0.329	78.7
Manicaland	0.108	0.102	0.322	0.108	0.115	0.461	−2.6
Mash Central	0.093	0.121	0	0.093	0.083	0.228	66.7
Mash East	0.207	0.121	0	0.207	0.21	0.737	95.6
Mash West	0.104	0.111	0.313	0.104	0.1	0.652	42.4
Mat North	0.149	0.118	0	0.149	0.142	0.486	78.3
Mat South	0.087	0.106	0.004	0.087	0.089	0.81	89.8
Midlands	0.079	0.135	0	0.079	0.076	0.61	93.3
Masvingo	0.113	0.132	0.006	0.113	0.124	0.22	43.2
Pseudo R^2^			0.045			0.002	
Mean bias			7.5			1.5	
*P*-value joint equality of means			0			0.995	

Notes: Balance check before and after PSM for observations for which 0.1 < e(X) < 0.9. Pseudo R2 indicates how well covariates explain treatment probability; a small value after matching indicates goodness of the matching technique [32]. A standardised absolute mean bias less than 5 after matching indicates effective matching [30]. A non-significant *p*-value for the joint mean equality test after matching is indicative of no significant differences between treatment and control groups after matching [11]

## Conclusion

The paper analysed the impact of food fortification on stunting in Zimbabwe. It has three major findings. Firstly, we found little evidence for gender differences in the knowledge or adoption of fortified foods. Secondly, we found that the adoption or knowledge of fortified foods reduces the proportion of stunted children in the household. Finally, we found that female headed households that adopt or know about fortified foods are more able to reduce the proportion of stunted children than their male counterparts. These results highlight the need for policy makers to actively promote fortification and biofortification programmes as such promotions can contribute to the reduction of stunting. More so, there is need to involve men in all fortification programmes to improve on their knowledge and appreciation of fortified foods and the associated benefits. Efficacy studies to gain insights in to the bioaccessibility of micronutrients from the fortified foods are essential to clearly understand the impact of fortification and biofortification on stunting.

### Policy implications

The results of this study are important for informing policy makers and programmers involved in fortification and biofortification programmes on the need to positively influence adoption of food fortification. The low knowledge on fortified (Table 2) reflects the need to integrate fortification and biofortification programmes into public and private policies, programmes, and investments. Policymakers should also give higher priority to the role of agriculture in improving health. At national level, there is need to include fortification and biofortification on the nutrition agenda. Moreover, food processors and other actors along the value chain must include fortified crops in their processed products.

## Data Availability

The datasets analysed during the current study are available from the Food and Nutrition Council of Zimbabwe (FNC) but restrictions apply to the availability of these data, which were used under a Memorandum of Understanding for the current study, and so are not publicly available. Data are however available from the authors upon reasonable request and with permission of FNC.
